# AIRE transcriptional condensates in central tolerance: a multiscale mechanistic perspective

**DOI:** 10.3389/fimmu.2026.1859704

**Published:** 2026-07-15

**Authors:** Jiejie He, Weiwei Xue, Jun Zhang, Yan Li

**Affiliations:** 1 Department of Gynecologic Oncology, Affiliated Hospital of Qinghai University & Affiliated Cancer Hospital of Qinghai University, Xining, Qinghai, China; 2 Department of Hepatopancreatobiliary Surgery, Affiliated Hospital of Qinghai University, Xining, Qinghai, China; 3 Department of Urology, Affiliated Hospital of Qinghai University, Xining, Qinghai, China

**Keywords:** AIRE, autoimmunity, central tolerance, medullary thymic epithelial cells, transcriptional condensates

## Abstract

Autoimmune regulator (*AIRE*) enables medullary thymic epithelial cells (mTECs) to express a broad but selective repertoire of tissue-restricted antigens (TRAs), thereby supporting central T-cell tolerance. Recent studies showing that *AIRE* forms transcriptionally active condensates provide a framework for understanding how chromatin recognition, enhancer-associated cofactor recruitment, three-dimensional genome organization and transcriptional elongation are coordinated during TRA induction. In this view, sparse TRA expression reflects the low probability that a given locus reaches a permissive chromatin and cofactor state, whereas high output may result from local enrichment of *BRD4*, P-TEFb, Mediator-associated co-activators and elongation machinery after activation. Recent findings on thymic mimetic cells, human thymic spatial organization and peripheral RORγt-associated antigen-presenting cells are discussed as related contexts, with particular attention to the current lack of direct evidence for mTEC-like *AIRE* condensate mechanisms outside mTECs. Together, these studies place *AIRE* condensates within a broader regulatory landscape of chromatin gating, transcriptional kinetics and mTEC state diversity.

## 1 Introduction

Central T-cell tolerance depends on exposing developing thymocytes to a broad but regulated spectrum of self-antigens within the spatial and temporal constraints of the thymic medulla. This antigenic exposure supports deletion of high-affinity autoreactive clones and contributes to the generation of regulatory T cells. *AIRE* is indispensable to this process; loss of *AIRE* disrupts central tolerance and pathogenic *AIRE* variants cause autoimmune polyendocrinopathy-candidiasis-ectodermal dystrophy, also known as autoimmune polyendocrine syndrome type 1 (APECED/APS-1), a disorder characterized by chronic mucocutaneous candidiasis, hypoparathyroidism, Addison’s disease, and related manifestations (1–3).

The best-characterized site of *AIRE* function is the mTEC compartment. mTECs ectopically express many genes normally associated with peripheral tissues, thereby providing antigenic substrates for negative selection and regulatory T-cell induction. Population-level transcriptomic studies show that thymic epithelial cells can transcribe up to 19,293 protein-coding genes and that mature medullary epithelial cells contain roughly 3,980 *AIRE*-positively regulated TRA genes (4). TRA expression, however, is not a uniform catalogue expressed by every mTEC. Individual antigens are detected in only ~1%-3% of mTECs, are organized into recurrent co-expression modules, and can reach markedly higher expression once activated in a single cell (4, 5). Thus, a central question is how *AIRE* distributes rare but high-amplitude antigen expression across the mTEC population.

This expression logic is difficult to explain by a simple model in which *AIRE* is recruited to isolated cis-regulatory elements and then activates nearby genes in a linear fashion. *AIRE* is not a canonical sequence-specific DNA-binding transcription factor. Instead, its activity appears to depend on chromatin state, enhancer-associated cofactor density, protein interaction networks, and transcriptional elongation. DNA topoisomerase I (TOP1), which relaxes transcription-associated DNA supercoiling and influences local chromatin topology, stabilizes parts of the *AIRE* interaction network: TOP1 perturbation weakens interactions between *AIRE* and multiple transcription-related partners (6). Genome-wide localization and proteomic studies further suggest that *AIRE* acts preferentially within regulatory-factor-rich chromatin environments rather than at isolated recognition sites (7).

**Figure 1:**
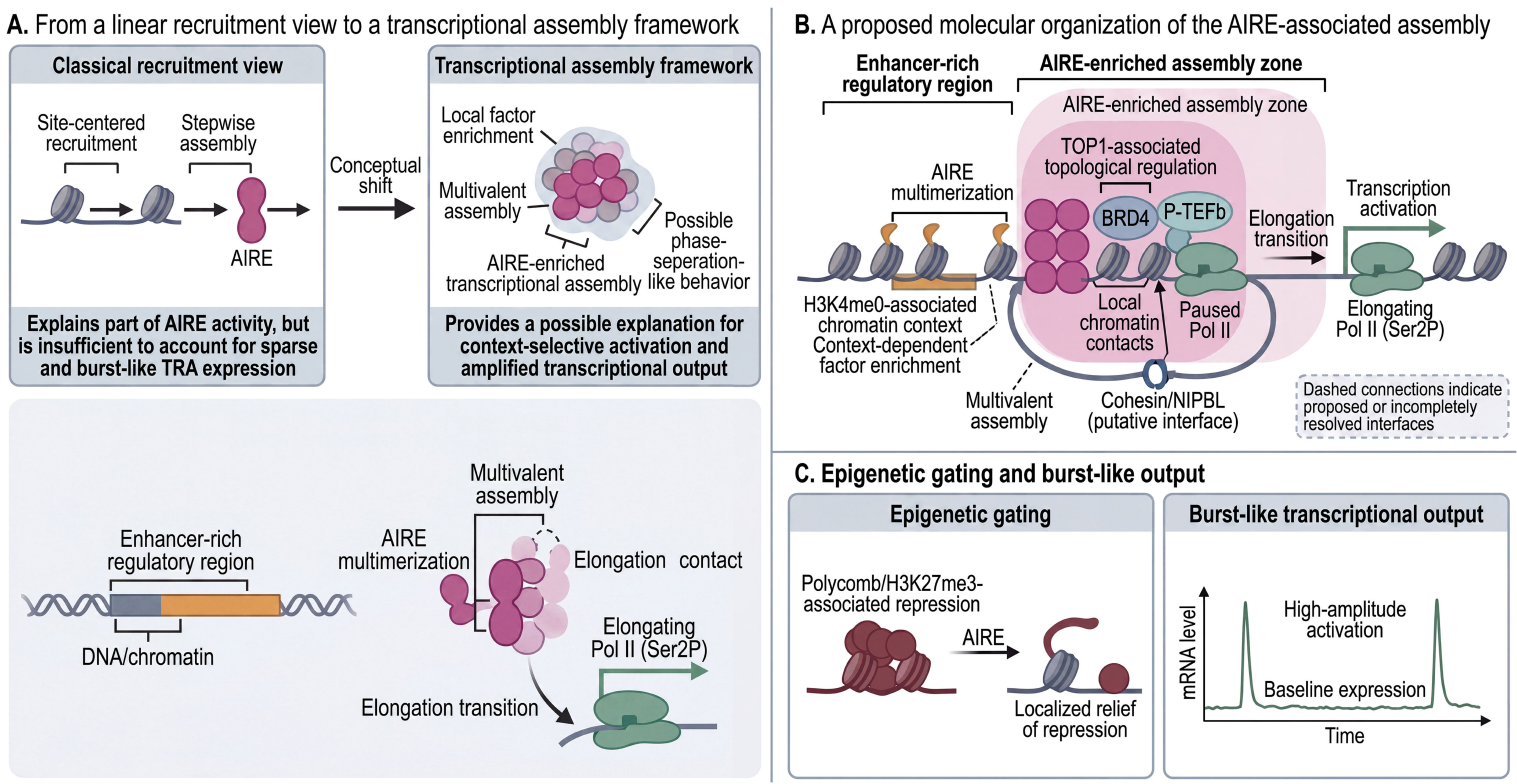
A working model for AIRE-associated transcriptional assembly in mTECs. **(A)** Conceptual transition from a site-centered recruitment view to an assembly-centered framework. The classical view emphasizes stepwise recruitment to target loci without implying canonical sequence-specific DNA binding by AIRE. The assembly-centered view proposes that AIRE acts through multivalent local enrichment, cofactor recruitment, and formation of AIRE-enriched transcriptional hubs within enhancer-rich regulatory environments. This framework may better explain context-selective activation, high-amplitude output, and sparse burst-like TRA expression. **(B)** Proposed molecular organization of an AIRE-associated assembly. AIRE multimerization is coupled to chromatin context, chromatin-contact interfaces, and recruitment of BRD4, P-TEFb, TOP1-associated topological regulators, cohesin/NIPBL, and paused RNA polymerase II. Dashed connections indicate proposed or incompletely resolved interfaces. **(C)** Model of epigenetic gating. Polycomb/H3K27me3-associated repression establishes a constrained but inducible state; productive AIRE assembly may help shift selected loci into high-output transcription, generating sparse but amplified TRA expression.

These findings have shifted attention from simple recruitment models toward *AIRE*-associated transcriptional assemblies. Earlier reviews have established the broader background of *AIRE* chromatin plasticity and negative selection (8, 9), *AIRE*-associated autoimmunity (10), mimetic-cell biology (11–13), and thymic-medulla remodeling (12, 14, 15). Building on this literature, the sections below integrate the controlled-condensate mechanism described by Huoh et al. (16) and its early interpretation in the field (17) with enhancer preference, chromatin looping, pause release, PRC2-associated restraint, and sparse yet amplified TRA expression in mTECs. This multiscale working model is summarized in **Figure 1**.

## 2 Central tolerance through the lens of transcriptional condensates

### 2.1 *AIRE* in the classical framework of central tolerance

In the classical framework, *AIRE* is the key regulator of ectopic TRA expression in mTECs and a core determinant of central immune tolerance (1, 18). Broader reviews of thymic tolerance and *AIRE*-dependent negative selection provide the classical background (9, 19). Recent work further emphasizes that central tolerance emerges from functionally diverse mTEC subsets rather than from one uniform epithelial population (15). Pathogenic *AIRE* mutations across diverse populations reinforce its upstream importance in preventing APS-1 (2, 3). Functional genomic studies show that *AIRE* controls a large but selective transcriptional program (4, 20). A frequently cited human mTEC transcriptomic study reported that sorted human mTEC populations analyzed by bulk RNA-seq expressed an average of 20,426 genes (21). This population-level result should therefore not be interpreted as evidence that individual *AIRE*-positive mTECs express approximately 20,000 genes; the analyzed populations were not restricted to *AIRE*-positive cells. At single-cell resolution, the defining feature of *AIRE*-dependent antigen display is sparse, heterogeneous, and modular TRA output rather than per-cell comprehensiveness.

### 2.2 A condensate-based view of *AIRE*-dependent TRA expression

*AIRE* function therefore appears to extend beyond recruitment to individual cis-elements. Available studies indicate that *AIRE* preferentially associates with extended chromatin intervals enriched for regulatory factors, including regions with super-enhancer-like features that may organize both nearby and distal transcription start sites (4, 6, 7). The relevant unit of regulation is therefore better viewed as a local regulatory environment rather than a single promoter or binding event.

The single-cell phenotype makes this point especially clear. An individual TRA is typically detected in only ~1%-3% of mTECs (22), yet *AIRE*-induced genes are not expressed as random noise. Approximately 51% of Aire-induced transcripts can be assigned to 19 recurrent co-expression clusters, each containing about 33–114 transcripts (median, 57), with strong within-cluster correlation and reduced clustering in the absence of Aire (23). Once an *AIRE*-dependent gene is activated, expression can be much higher than the population mean; in one analysis of 174 individual mTECs, detected *AIRE*-induced genes were expressed at approximately 16-fold higher levels than the population average (4). These features point to a thresholded system in which rare activation events are locally amplified.

Super-enhancer biology and transcriptional condensate models provide a useful framework for interpreting this threshold-like behavior. Super-enhancers are extended enhancer clusters with high co-activator density; by concentrating *BRD4*, MED1, and other transcriptional regulators, they may increase both the probability and amplitude of transcription from selected loci (24, 25). Huoh et al. showed that *AIRE* can assemble enhancer-associated condensates that promote local transcriptional activity and connect genomic loci across chromosomes (16). This controlled assembly requires coordinated action of the CARD, PHD1, and C-terminal tail (CTT) domains. These findings connect *AIRE* condensate formation to chromatin topology, elongation control, and single-cell TRA output without requiring every *AIRE*-bound region to be interpreted as a condensate.

## 3 Structural basis of *AIRE* condensate assembly

### 3.1 Domain architecture of *AIRE* and its assembly interfaces

*AIRE* has a modular architecture that supports multivalent interactions and regulated nuclear assembly. Human *AIRE* is approximately 545 amino acids long and contains an N-terminal caspase recruitment domain (CARD), a SAND domain, two plant homeodomain (PHD) zinc fingers, and a C-terminal tail (CTT), separated by low-complexity regions that contribute to nuclear localization, chromatin recognition, and partner recruitment (26). The CARD provides a self-assembly interface that supports *AIRE* nuclear puncta formation; mutations that disrupt CARD-mediated assembly impair nuclear localization and transcriptional output (27, 28). CARD filament mutants such as R16A/E18A and D35A/D37A lose nuclear foci and transcriptional activity, indicating that polymerization and function are tightly coupled in these experimental systems (27). Pathogenic APS-1/APECED variants across conserved domains, including CARD and PHD1, further link structural integrity to downstream TRA regulation (29, 30).

Recent mechanistic work places these domains into a coherent assembly model. *AIRE* can form enhancer-associated condensates that increase local transcriptional activity and connect loci across chromosomes (16). CARD-mediated polymerization supplies the assembly interface, PHD1 contributes chromatin-state engagement, and the CTT binds co-activators such as CBP/p300 to bias *AIRE* toward appropriate regulatory sites. This domain cooperation explains why *AIRE* assembly must be controlled rather than simply maximized: productive condensates require both multimerization capacity and correct chromatin/cofactor context.

### 3.2 Histone sensing and chromatin interactions underlying targeting

*AIRE* targeting is therefore best understood as chromatin-context sensing rather than canonical sequence-specific DNA recognition (26). *AIRE*-PHD1 preferentially recognizes unmethylated H3K4 (H3K4me0), with reported binding affinities in the micromolar range (Kd approximately 5 μM) (31, 32). *In vivo* and genome-wide analyses indicate that the histone H3-binding module is required for *AIRE*-dependent gene regulation and tolerance induction, although it almost certainly operates with additional targeting mechanisms (33). Functionally, PHD1 links *AIRE* to relatively silent or insufficiently activated loci, providing an epigenetic entry point for TRA activation in mTECs.

Histone recognition is only one layer of targeting. *AIRE* recruitment is also coordinated with DNA damage repair-related pathways. DNA-dependent protein kinase (DNA-PK) is a nuclear serine/threonine kinase composed of the catalytic subunit DNA-PKcs and the DNA-end-binding Ku70/Ku80 heterodimer, and it acts as a core effector of non-homologous end joining and the broader DNA double-strand break repair response. H3K4me0 and DNA-PK together promote *AIRE* recruitment to TRA loci, and loss of DNA-PKcs disrupts *AIRE* assembly and transcriptional activity at chromatin and episomal targets (34). Recent work suggests that Z-DNA-associated features may contribute to *AIRE* target selection during thymic T-cell tolerization (35). Conversely, although *AIRE* contains a SAND domain, mechanistic work indicates that this domain lacks residues required for direct canonical DNA recognition (16). The emerging picture is therefore a layered targeting system in which histone state, DNA structure, DNA-PK-associated repair pathways, enhancer context and cofactor availability jointly influence where productive *AIRE* assemblies may form.

### 3.3 Gating multimerization and condensate assembly

CARD-mediated polymerization provides the structural basis for *AIRE* nuclear puncta, but productive condensate formation depends on how this polymerization is gated. Broad PHD1-mediated engagement with H3K4me0 may keep *AIRE* distributed across many potential genomic sites, limiting inappropriate polymerization. In contrast, CTT interaction with co-activators such as CBP/p300 biases *AIRE* toward enhancer-rich environments and permits controlled CARD-dependent assembly (16). Thus, CARD supplies assembly capacity, PHD1 contributes chromatin dispersal and targeting, and the CTT links *AIRE* to co-activator-rich regulatory neighborhoods. This division of labor explains how *AIRE* can combine broad genomic reach with selective local activation.

**Figure 2:**
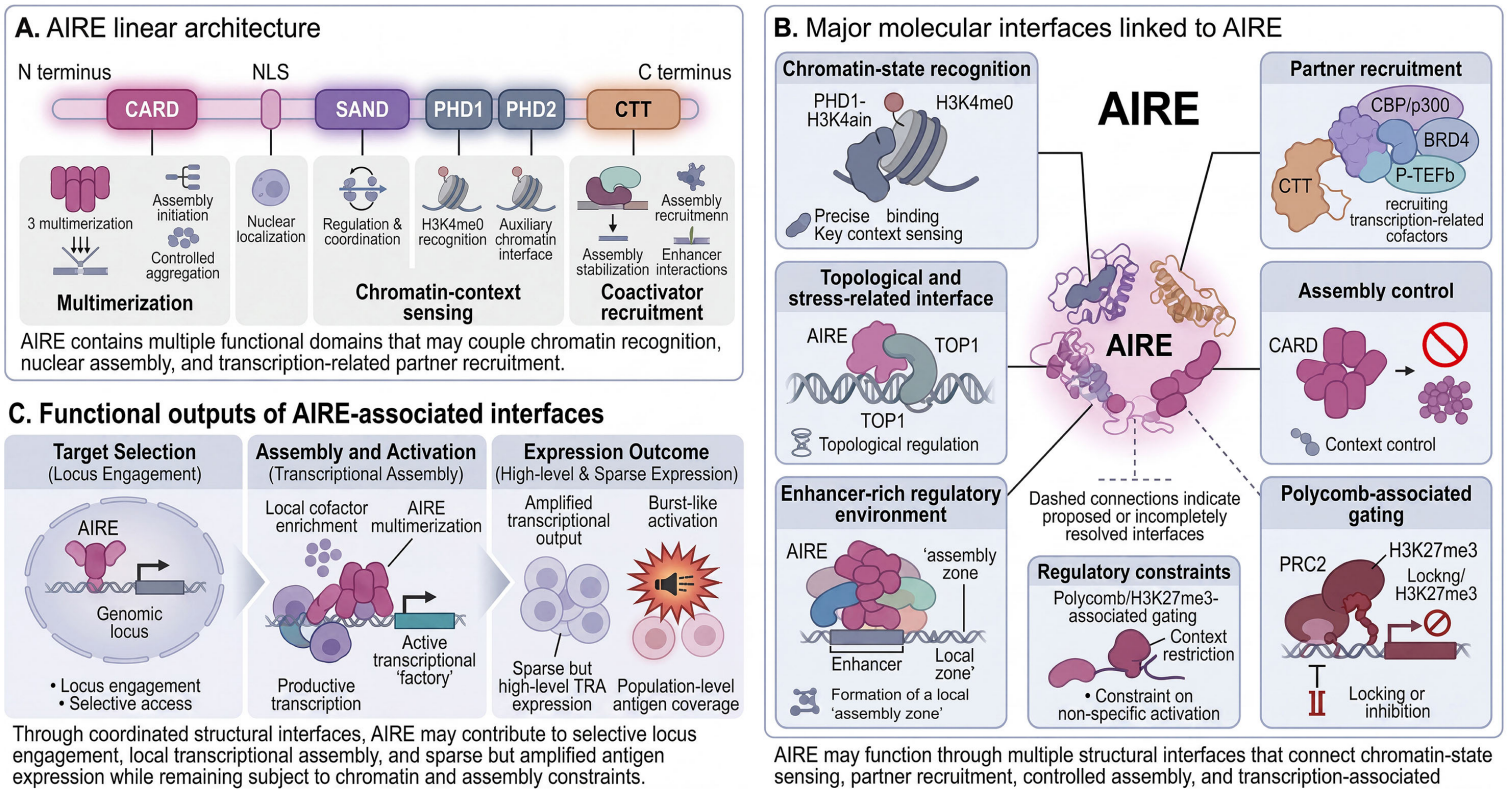
Structural interfaces of AIRE and their functional implications in transcriptional assembly. **(A)** Schematic overview of AIRE domain architecture, including CARD, nuclear localization signal (NLS), SAND, PHD1, PHD2, and C-terminal tail (CTT) domains. These domains support multimerization, nuclear localization, chromatin-context sensing, and cofactor recruitment. **(B)** Major molecular interfaces linked to AIRE, including H3K4me0 recognition, CBP/p300, BRD4, P-TEFb, TOP1-associated topology, and CARD-dependent assembly control. Dashed connections indicate proposed, indirect, or incompletely resolved interfaces. **(C)** Functional outputs of these interfaces include selective chromatin engagement, local transcriptional assembly, elongation release, and sparse but amplified TRA expression.

Assembly is coupled to transcriptional kinetics. *AIRE* promotes the transition from promoter-proximal pausing to productive elongation by engaging *BRD4* and positive transcription elongation factor b (P-TEFb), a CDK9-containing kinase complex that releases paused RNA polymerase II into elongation (36). This coupling depends on post-translational modification of *AIRE* within the CARD region and is directly linked to tolerance induction. APECED-associated mutations can disrupt *AIRE*-P-TEFb coupling and impair downstream transcriptional output (37). Thus, *AIRE* condensates may be viewed as regulatory states that integrate partner recruitment, chromatin context, and elongation control rather than as passive nuclear clusters (**Figure 2**).

## 4 *AIRE* condensates in super-enhancer contexts

### 4.1 Preference for super-enhancer occupancy in mTECs

Functionally relevant *AIRE* condensates are most likely to occur within defined regulatory contexts rather than as nonspecific nuclear aggregates. ChIP-seq profiling of mTEC^hi^ cells identified 42,124 *AIRE*-occupied sites, with more than 75% overlap between biological replicates (6). Their distribution supports the view that *AIRE* enrichment is shaped less by classical sequence recognition than by chromatin environments with locally accumulated regulatory factors (38, 39).

Super-enhancer analyses support this interpretation. H3K27ac-based ROSE analysis identified 1,170 super-enhancer-like regions in mTEC^hi^ cells, with an average length of approximately 30 kb. *AIRE* density was higher within these regions than within conventional enhancers (6, 40), and loss of *AIRE* reduced H3K27ac density and chromatin accessibility at the same regulatory regions. In comparative ATAC-seq analyses, the accessibility difference between Aire+/+ and Aire-/- mTEC^hi^ cells at super-enhancers was highly significant (P = 6.6 x 10^-52^) (6). These data support a close association between *AIRE* and high-density regulatory platforms, although they do not by themselves prove that every *AIRE*-occupied super-enhancer forms a condensate.

These enhancer-rich regions may provide a favorable environment for assembly of the *AIRE* partner network. *AIRE*-associated complexes include transcriptional, chromatin, topological, and RNA-processing components rather than a single uniform cofactor set. TOP1 appears to act as an important stabilizing component of the *AIRE*-associated interaction network: shRNA-mediated TOP1 knockdown weakens interactions between *AIRE* and many transcription-related partners while having a smaller effect on splicing-related factors. TOP1 and γH2AX also show partial co-localization with *AIRE* at enhancer-rich regions in mTEC^hi^ cells, suggesting that these loci may integrate *AIRE* enrichment, partner assembly, and local topological regulation (6, 41–43).

### 4.2 Three-dimensional genome remodeling and enhancer–promoter coupling

*AIRE*-associated super-enhancer regions also connect *AIRE* function to three-dimensional genome organization. In general models of genome organization, loop anchors and topological hubs can increase enhancer-promoter contact frequency and support transcriptional output. Mechanistic and modeling studies of loop extrusion provide the broader physical context for this idea (44, 45). Developmental and *in vitro* studies further support the physical plausibility of cohesin- or condensin-driven extrusion (46, 47). In mTECs, *AIRE*-associated super-enhancers are linked to local and distal transcription start sites, and *AIRE* can reshape chromatin looping by reducing CTCF occupancy at domain boundaries while favoring cohesin accumulation on super-enhancers (6, 7). Thus, the general topology literature supplies context, whereas the mTEC-specific inference rests on *AIRE* super-enhancer and looping studies.

The interaction between *AIRE* and the cohesin loader NIPBL provides one mechanistic route into genome folding. *AIRE* can recruit NIPBL to super-enhancer-rich regions and thereby promote chromatin looping and activation of distant loci (7). This observation supports a model in which *AIRE*-containing assemblies nucleate within enhancer-dense domains and couple co-activator recruitment to local loop remodeling. The ability of *AIRE* condensates to bridge loci on different chromosomes (16) suggests that *AIRE*-dependent activation may operate across multiple spatial scales, from enhancer-promoter proximity to broader nuclear-contact organization. How these spatial scales are used at individual TRA loci remains unclear.

### 4.3 Control of transcriptional kinetics and pause release

The condensate model must also account for transcriptional kinetics, not only chromatin proximity. *AIRE*-regulated genes are enriched for promoter-proximal paused RNA polymerase II, and *AIRE* can promote release of paused polymerase into productive elongation (48). Promoter-proximal pausing occurs when RNA polymerase II initiates transcription but remains transiently restrained near the transcription start site until elongation factors are recruited. Many *AIRE* targets reside in relatively inactive chromatin and may therefore require both enhancer engagement and a kinetic switch. *AIRE* provides such a switch by engaging *BRD4* and positive transcription elongation factor b (P-TEFb), a CDK9-containing complex that phosphorylates the pause-release machinery and supports productive elongation (36, 49).

This kinetic view helps explain how sparse detection can coexist with high output at activated loci. Sparse detection may reflect the low probability that a given TRA locus reaches a permissive combination of chromatin accessibility, enhancer contact, *AIRE* nucleation and cofactor availability in an individual mTEC. Once this threshold is crossed, local enrichment of *AIRE*, *BRD4*, P-TEFb, MED1-associated co-activator assemblies, and paused polymerase could increase the probability of pause release, extend the duration of an active burst, and permit repeated rounds of polymerase re-initiation from the same locus (36, 48). In this scenario, a rare activation event is converted into a high-output transcriptional episode, while most loci in most cells remain below threshold. Direct measurements of nascent transcription, *AIRE* residence time, enhancer-contact persistence, and burst kinetics in primary mTECs will be needed to test this model.

## 5 Epigenetic gating and stochastic TRA expression

### 5.1 Polycomb-associated repression and an inducible context

Sparse TRA expression is shaped by the repressive chromatin landscape on which *AIRE* acts. In general terms, epigenetic gating describes how access to the genome is controlled by DNA methylation and the associated histone modifications, which together determine whether a given locus is permissive or refractory to transcription. Here, epigenetic gating refers to a repressive but inducible chromatin state that shapes the probability of TRA activation at individual loci. Many *AIRE*-dependent TRA loci are marked by Polycomb repression, especially H3K27me3, the trimethylation of histone H3 lysine 27 (50–52). *AIRE*-dependent transcription therefore emerges not from a uniformly permissive genome, but from loci that are restrained before activation.

PRC2, the enzyme complex that deposits H3K27me3, provides functional support for this gating model. Developmental ChIP-seq analyses indicate that tissue-specific genes in mTECs acquire H3K27me3 during transition from immature to mature states, with particular enrichment at genes induced during late maturation (53). Genetic and recent mechanistic studies further indicate that PRC2 shapes both ordered stochastic expression of Aire targets and development of mimetic cells, although the exact molecular handoff between Polycomb repression and *AIRE* condensate activation remains incompletely resolved (52, 54). PRC2 may therefore shape the probability of *AIRE*-dependent activation rather than functioning as a simple binary antagonist of *AIRE*.

The gate is also dynamic. The H3K27 demethylase Kdm6b is required for mTEC homeostasis and function; loss of Kdm6b reduces mature mTEC numbers, impairs TRA expression, and compromises immune tolerance (55). These findings suggest that *AIRE*-dependent activation is shaped by the balance between Polycomb-mediated methylation and demethylation rather than by a fixed repressive state. Changes in chromatin-modifying enzymes may therefore shift the probability that an *AIRE*-associated assembly becomes transcriptionally productive.

### 5.2 mTEC subset diversity and *AIRE*-dependent regulatory modes

*AIRE* operates within a heterogeneous epithelial compartment. Single-cell studies support a continuum of mTEC differentiation states and regulatory modules rather than a single terminal population (56–58). Within this continuum, *AIRE* intersects with other lineage regulators, including FEZF2/Fezf2 (FEZ family zinc finger 2), that contribute to the overall self-antigen landscape (59). Consequently, the same *AIRE*-centered machinery may produce different outputs depending on cell state, chromatin accessibility, and cofactor availability.

*AIRE*-dependent output is therefore not deployed identically across all mTEC subsets. Integrated single-cell transcriptomic and DNA methylomic analyses resolve mature mTECs into populations with overlapping but non-identical antigen-presentation programs (56). Some subsets appear enriched for strong Aire-associated promiscuous gene expression, whereas others preferentially deploy alternative lineage-defining programs. Single-cell heterogeneity therefore reflects structured deployment of antigen-expression programs across differentiated epithelial states rather than random noise around a homogeneous cell type.

Mimetic cells provide another layer of this cell-state dependence. Thymic tuft-cell studies first established that the medulla contains epithelial subsets with specialized peripheral-like features (60, 61). Broader single-cell and enhancer studies then showed that mTECs can co-opt lineage-defining transcription-factor programs to generate tissue-like mimetic states, including gut-, muscle-, neuroendocrine-, and other lineage-associated programs (62–64). These subsets suggest that tolerance is built not only through stochastic activation of isolated TRAs but also through controlled deployment of tissue-like transcriptional modules. *AIRE* may contribute to this architecture, but current evidence does not support treating it as the universal master regulator of all mimetic identities.

### 5.3 A gating model for stochastic activation and modular output

**Figure 3:**
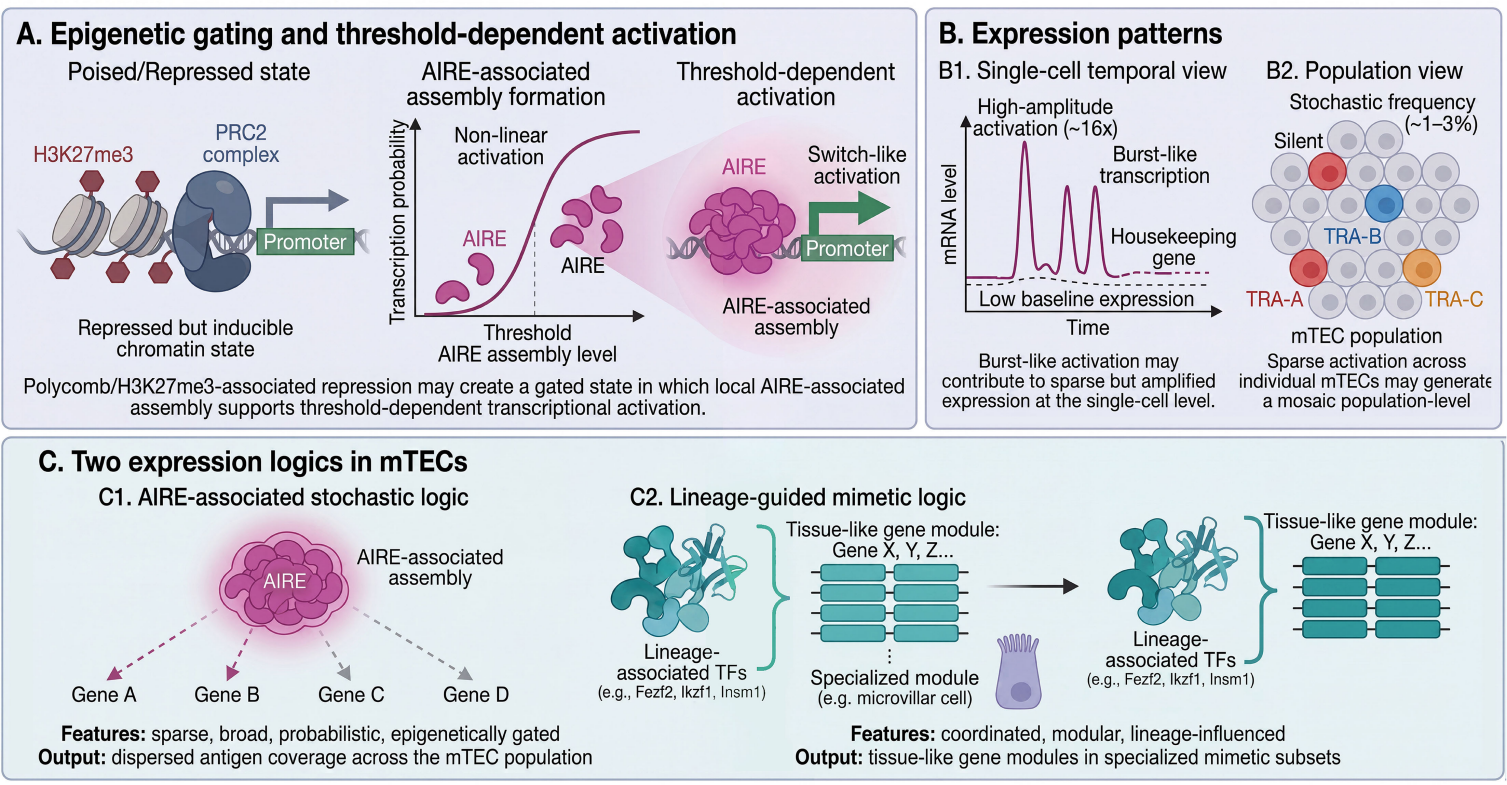
Epigenetic gating and complementary expression logics underlying mTEC antigen display. **(A)** Epigenetic gating model. PRC2/H3K27me3-associated repression maintains many TRA loci in a constrained but inducible state. Productive AIRE-associated assembly may permit selected loci to cross an activation threshold. **(B)** Expression logic. At single-cell resolution, AIRE-related targets may show low baseline expression with burst-like activation; at the population level, sparse activation across different mTECs generates mosaic TRA coverage. **(C)** Complementary mTEC programs. AIRE-associated stochastic activation and lineage-guided mimetic programs provide distinct but complementary modes of self-antigen display.

Together, PRC2-associated restraint and *AIRE*-dependent activation yield a testable gating model. Most tissue-specific loci remain in repressed or low-probability states across the mTEC population. A subset of cells transiently acquires the chromatin context, cofactor availability, and *AIRE* assembly state needed for productive elongation. Once that threshold is crossed, transcription is locally amplified, producing high-level expression of a limited set of TRA modules. This model helps explain why TRA expression is rare, modular, and high-amplitude at single-cell resolution (4, 23) and is summarized conceptually in **Figure 3**.

Pathological failure could occur on either side of this gate. If Polycomb-associated repression is too rigid or improperly resolved, *AIRE* may not gain productive access to selected loci, reducing antigen coverage. Conversely, if *AIRE* multimerization, partner recruitment, or elongation control is impaired, loci that are otherwise poised for activation may fail to enter high-output states. In both cases, the defect is not simply a lower number of expressed genes; it is a breakdown in the probabilistic architecture that distributes self-antigen display across the mTEC population.

## 6 Single-cell atlases and the emergence of mimetic cells

### 6.1 mTEC heterogeneity and the definition of mimetic cells

Single-cell omics has transformed mTEC heterogeneity from a descriptive observation into a map of cell states, trajectories, and antigen-output programs (64, 65). Functional studies further indicate that distinct mTEC subsets cooperate in central tolerance rather than simply representing redundant versions of the same cell state (15). The medullary compartment contains specialized subsets that differ in maturation stage, transcription-factor usage, and antigenic repertoire. This atlas places *AIRE* activity within a diversified epithelial compartment rather than within a uniform terminal cell type.

Mimetic cells include tuft-like, muscle-like, neuroendocrine-like, epithelial, and other specialized subsets that reproduce selected peripheral tissue features within the thymus (62, 64). The emergence of thymic mimetic cells as a distinct conceptual category has also been highlighted in field commentary (66). Developmental conversion of embryonic thymic epithelial states provides an additional route by which antigen-displaying medullary programs emerge (67). These programs appear to be actively specified thymic epithelial states rather than passive leakage of peripheral identity (62, 64). They provide a second organizing principle for tolerance: tissue-like modules can complement stochastic TRA activation by presenting self-information in coordinated cellular contexts.

Functionally, these subsets appear to cooperate rather than simply duplicate one another. CCL21-associated mTECs support thymocyte migration and medullary organization, tuft-like mTECs shape cytokine environments, and other mimetic lineages contribute distinct antigenic repertoires (68, 69). Central tolerance can therefore be viewed as an emergent property of the medullary network. *AIRE* remains a major axis of antigen diversification, but its output is embedded within a broader multicellular architecture.

### 6.2 Network constraints and allocation of mimetic lineages

Mimetic cells also reveal a regulatory constraint. mTECs must deploy lineage-defining transcriptional logic strongly enough to generate tissue-like antigenic features, but not so strongly that they undergo wholesale lineage conversion. Ikaros (Ikzf1) has emerged as a principal regulator of Aire-positive mTEC homeostasis and mimetic-cell diversity, linking thymic epithelial network architecture to allocation of mimetic states (70).

Insm1 provides another example of this developmental control. Insm1 deficiency impairs mTEC development and immune tolerance, suggesting that appropriate epithelial-state formation is a prerequisite for correct deployment of *AIRE*-dependent and *AIRE*-adjacent programs (71). Thus, transcriptional output attributed to *AIRE* cannot be interpreted independently of the developmental circuitry that establishes the cellular context in which *AIRE* acts.

Dependence on *AIRE* also differs across mimetic lineages. Lineage-defining transcription factors are required for accumulation of specific mimetic subsets, whereas *AIRE* appears to modulate only part of the associated transcriptional repertoire (62, 63). New developmental studies strengthen this distinction. RUNX1 deficiency reveals thymic alveolar type 2 epithelial mimetic cells, and RUNX3 has been linked to Aire+ mTEC development, tissue-specific antigen expression, and central tolerance (72, 73). These data support a division of labor: lineage factors allocate epithelial state space, while *AIRE* contributes a condensate-linked activation module within selected states rather than acting as a universal master regulator of all mimetic programs.

### 6.3 Cross-species conservation of mimetic cells and human models

Mimetic cells can be viewed as a modular tolerance strategy that generates localized tissue-like antigenic domains within the thymus. Cross-species studies support conservation of this broad logic while also identifying species-specific elements (13, 68). Recent spatial mapping of the human thymus further supports this view by localizing lineage-associated transcription factors within rare mimetic epithelial populations (65). The implication is that tolerance benefits from architectural diversification of antigen display, not merely from increasing the total number of genes expressed.

**Figure 4:**
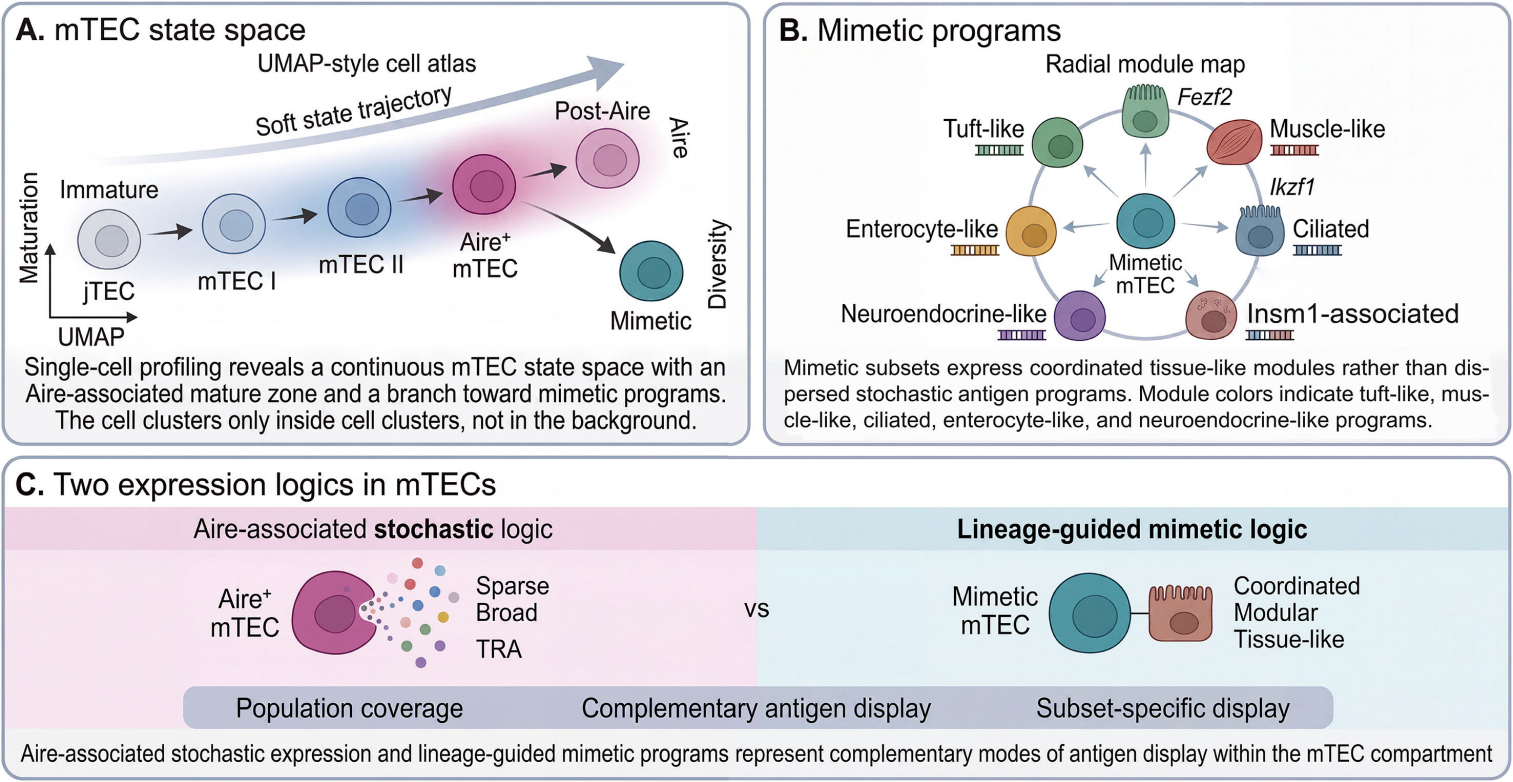
Single-cell atlas of mTEC heterogeneity and mimetic programs in central tolerance. **(A)** Schematic mTEC state space inferred from single-cell profiling, spanning immature/junctional TECs, intermediate states, mature Aire-expressing mTECs, post-Aire states, and mimetic lineages. **(B)** Representative mimetic programs, including tuft-like, enterocyte-like, muscle-like, neuroendocrine-like, and ciliated states, with selected regulators such as Fezf2, Ikzf1, and Insm1. **(C)** Conceptual comparison between sparse AIRE-associated stochastic TRA activation and coordinated lineage-guided mimetic programs. These strategies may jointly expand the breadth and organization of self-antigen presentation in the thymic medulla.

Experimentally tractable human systems now provide platforms to test these ideas directly. Reviews of pluripotent stem-cell differentiation into thymic epithelial cells frame organoid systems as potential tools for modeling APECED-relevant thymic biology (74). Human pluripotent stem cell-derived thymic epithelial models recapitulate aspects of human thymic epithelial development and multilineage specification (75, 76). Adult and engineered thymic epithelial organoid systems provide complementary platforms for modeling thymic epithelial function and T-cell development (77–79). These systems may help determine how *AIRE* activity, mimetic-state allocation, and chromatin context are coordinated in human cells, where species differences limit direct extrapolation from mouse models (**Figure 4**).

## 7 Perspectives: peripheral *AIRE* biology and open questions

Peripheral *AIRE* biology is best discussed as a related but distinct area of tolerance research (80, 81), rather than as a direct extension of the mTEC condensate mechanism. Direct evidence for *AIRE* condensate assembly, enhancer-local transcriptional activation, and interchromosomal bridging is currently strongest in mTECs and reconstituted systems (16). Peripheral *AIRE*- or RORγt-related antigen-presenting cells may share selected tolerance functions with mTECs, but their lineage identity, antigen sources, and regulatory environments differ. They are therefore best viewed as distinct APC states whose possible relationship to *AIRE* condensates remains an open question.

**Table 1** summarizes peripheral *AIRE*- or RORγt-associated tolerance settings that are relevant to, but mechanistically distinct from, mTEC *AIRE* biology. Across these studies, the shared theme is tolerogenic antigen presentation outside the classical thymic epithelial compartment; however, the evidence mainly supports context-dependent APC states, microbiota- or food-antigen–linked tolerance, or RORγt-associated immune regulation rather than direct reuse of the mTEC *AIRE*-condensate program. At present, none of these peripheral systems has demonstrated *AIRE*-containing enhancer condensates, interchromosomal bridging, or stochastic TRA activation through an mTEC-like pause-release mechanism. Therefore, these cell populations should be discussed as comparative tolerance contexts rather than as proven extensions of the mTEC condensate model.

**Table 1:** Evidence boundaries for peripheral and non-mTEC AIRE-related tolerance contexts.

Cell type or population	Main context	Reported immune role	Evidence boundary for *AIRE* condensates	Representative references
Classical eTACs	Secondary lymphoid organs	Deletion or functional inactivation of autoreactive CD4^+^ T cells in selected models	Classical eTACs were originally defined as extrathymic Aire-expressing APCs, but their lineage identity and stability are context dependent. They should not be treated as simple peripheral counterparts of mTECs, and direct evidence for *AIRE* condensates has not been demonstrated.	(82–84)
Aire-protein ILC3-like cells	Lymph nodes	Local antigen presentation and immune regulation	These cells support the existence of an APC state with detectable Aire protein, but current evidence does not establish broad mTEC-like TRA output or nuclear *AIRE* condensate assembly.	(85)
AmDCs/Janus-like populations	Peripheral lymphoid tissues	Tolerogenic APC states and peripheral immune restraint	Single-cell multiomic studies define Aire-positive APC states with dendritic-cell-like and epithelial-homology features. However, lineage relationships and mechanistic *AIRE* dependence remain under refinement, and direct *AIRE* condensate evidence is lacking.	(86)
R-eTACs and Thetis cells	Gut-associated and early-life intestinal tolerance	Treg-dependent tolerance to gut microbiota and dietary antigens	These populations illustrate microbiota-, food-antigen-, and developmental-context-dependent tolerance. Their functions should not be interpreted as a general self-TRA display program or as evidence for mTEC-like *AIRE* condensate reuse.	(87–90)
RORγt-positive DCs/related intestinal APCs	Oral antigen and intestinal tolerance	Peripheral regulatory T-cell induction in response to oral and intestinal antigens	These cells are important for peripheral tolerance, but available studies do not show *AIRE*-dependent enhancer condensates, stochastic TRA expression, or pause-release mechanisms analogous to those in mTECs.	(91, 92)
Related RORγt APC/ILC3 programs	Mucosal and peripheral immune settings	Treg induction, oral tolerance, antifungal defense, and cell-state support	These programs show the functional diversity of peripheral tolerance APCs. *AIRE* transcript or protein involvement varies by system, and direct evidence for *AIRE* condensate-based regulation has not been demonstrated.	(93–95)

B-cell contexts raise a similar interpretive issue. Thymic B cells can participate in central tolerance through antigen capture, B-cell receptor-dependent antigen presentation, and self-antigen-driven class switching, but they are not epithelial cells and should not be treated as mTEC replicas (96–98). Recent commentaries further emphasize thymic B cells as important contributors to T-cell tolerance (99). B-cell-mediated tolerance can also be relevant in disease-associated antigen settings, as illustrated by tolerance to the neuromyelitis optica autoantigen AQP4 (100). Age-related changes in thymic B-cell Aire expression provide an additional context, but they do not establish mTEC-like condensate regulation (101, 102). Germinal center B-cell *AIRE* biology is more distant from the mTEC paradigm because it relates to antibody diversification checkpoints rather than population-wide TRA display (103). Future work should test peripheral *AIRE* mechanisms directly through live-cell imaging, chromatin profiling, perturbation of *AIRE* assembly interfaces, and single-cell nascent-transcription measurements before extending the condensate model beyond mTECs.

## 8 Conclusion

Current evidence supports a model in which *AIRE* coordinates tolerogenic transcriptional assemblies in mTECs rather than acting as a conventional sequence-specific transcription factor. The strongest mechanistic evidence links *AIRE*-containing enhancer assemblies with three-dimensional genome contacts, pause-release kinetics and PRC2/H3K27me3-associated chromatin gating, while emerging work on Z-DNA-associated targeting adds an additional layer to *AIRE* locus selection. This view helps explain how rare TRA activation events can become high-output antigen-expression episodes while preserving the single-cell sparsity that characterizes mTEC antigen display. Recent studies of spatial human thymus organization, RUNX-dependent mimetic-cell programs, RORγt-associated peripheral APCs, thymic B cells, and germinal center B cells extend the biological context of *AIRE*-related tolerance, but they do not yet demonstrate shared condensate-based TRA regulation. Future work should directly measure *AIRE* residence time, condensate composition, enhancer-contact dynamics and nascent transcription in defined mTEC states and, where relevant, in peripheral *AIRE*-related cells.
